# Reducing ether lipids improves Drosophila overnutrition-associated pathophysiology phenotypes via a switch from lipid storage to beta-oxidation

**DOI:** 10.1038/s41598-022-16870-4

**Published:** 2022-07-29

**Authors:** Christie Santoro, Ashley O’Toole, Pilar Finsel, Arsalan Alvi, Laura Palanker Musselman

**Affiliations:** Department of Biological Sciences, Binghamton University, Binghamton, NY 13902 USA

**Keywords:** Biochemistry, Molecular biology

## Abstract

High-calorie diets increase the risk of developing obesity, cardiovascular disease, type-two diabetes (T2D), and other comorbidities. These “overnutrition” diets also promote the accumulation of a variety of harmful lipids in the heart and other peripheral organs, known as lipotoxicity. However, the mechanisms underlying lipotoxicity and its influence on pathophysiology remain unknown. Our study uses genetics to identify the role of ether lipids, a class of potential lipotoxins, in a Drosophila model of overnutrition. A high-sugar diet (HSD) increases ether lipids and produces T2D-like pathophysiology phenotypes, including obesity, insulin resistance, and cardiac failure. Therefore, we targeted ether lipid biosynthesis through the enzyme dihydroxyacetonephosphate acyltransferase (encoded by the gene *DHAPAT*). We found that reducing *DHAPAT* in the fat body improved TAG and glucose homeostasis, cardiac function, respiration, and insulin signaling in flies fed a HSD. The reduction of *DHAPAT* may cause a switch in molecular signaling from lipogenesis to fatty acid oxidation via activation of a PPARα-like receptor, as bezafibrate produced similar improvements in HS-fed flies. Taken together, our findings suggest that ether lipids may be lipotoxins that reduce fitness during overnutrition.

## Introduction

Obesity, type two diabetes (T2D), and cardiovascular disease have strong correlations with overnutrition, but the mechanisms by which obesity can increase the risk for the associated pathophysiology are not well understood. There are healthy obese individuals and those that become insulin resistant at a low BMI, suggesting that obesity itself is a correlate, not a cause, of pathophysiology. Obesity influences metabolic pathways including lipogenesis, lipolysis, and insulin signaling and leads to widespread accumulation of a range of lipid species^1,2^, known as lipotoxicity; however, the mechanisms of lipotoxicity and how it is linked to pathophysiology remain unknown. Scientists have proposed a maximum adipose expandability model, suggesting that habitual overnutrition leads to the complete occupation of adipose fat storage. This leaves newly synthesized free fatty acids and their derivatives to accumulate as toxic lipids or “lipotoxins” in peripheral tissues, such as the heart^3^. Evidence supporting this maximum adipose expandability model comes from studies showing that expansion of lipid stores surgically, pharmacologically, or genetically, improves metabolic disease^4,5^. In addition, this model explains medical anomalies in humans, such as the insulin resistance observed in lean lipodystrophic patients and the cohorts of metabolically healthy, obese subjects^6–8^. While the identity and function of the lipotoxins associated with overnutrition are not well understood, previous studies have associated a number of lipid classes, including diglycerides (DAG), diglyceride ethers (DAGE), acyl-carnitines (AC), triglycerides (TAG), ceramides, and free fatty acids (FFA), with obesity-associated pathophysiology^9,10^.

A number of animal models have been used to explore the biochemistry of lipotoxicity. Three organelles that are central to this process are the mitochondrion and the peroxisome, which metabolize lipids, and the lipid droplet, where lipids are stored. Mitochondrial fatty acid oxidation seems to protect against lipotoxicity, because mice that overexpress beta-oxidation enzymes exhibit improved glucose and insulin homeostasis on high-sugar or high-fat diets^11,12^. Mitochondrial uncoupling also dissipates excess energy, as uncouplers like BAM15 can also protect against insulin resistance in rodents and humans^13^. Peroxisomal proliferator drugs also improve lipotoxicity and insulin sensitivity^14,15^ and activation of the mammalian peroxisomal proliferator activated receptor PPARα can increase lipid catabolism^16^. Finally, lipid storage in droplets can be protective^17,18^ or deleterious in mammals^1,19^, depending on the organ and type of lipids that accumulate.

Drosophila is useful to understand pathophysiological mechanisms because flies recapitulate the overnutrition-associated pathophysiology phenotypes found in mammalian T2D models, including hyperglycemia, hyperinsulinemia, cardiomyopathy, insulin resistance, and increased TAG and free fatty acids^20–24^. Lipid metabolic pathways are highly conserved between flies and mammals^25^ and Drosophila offers easy genetic and dietary manipulation to efficiently study pathophysiology phenotypes^26–28^. Several studies have used Drosophila genetics to model lipotoxicity-associated metabolic pathways that are conserved in mammals^23,29–31^.

Tissue-specific studies in the Drosophila fat body suggest a critical and central role for this tissue in the control of lipotoxicity. The fat body is functionally like human adipose tissue, storing fat during nutrient excess and hydrolyzing stored fat for energy during starvation. Fat body-specific genetic manipulations are consistent with the maximum adipose expandability model. Flies in which fat body lipogenesis is reduced via knockdown of the carbohydrate response element binding protein^20,32^ fatty acid synthase^33^, or stearoyl-CoA desaturase 1^34^ exhibit reduced TAG accumulation accompanied by impaired sugar tolerance and increased pathophysiology phenotypes. In another study, high-fat-induced cardiac pathophysiology phenotypes improved after fat body-specific knockdown of Drosophila *target of rapamycin* (*dTOR*), accompanied by a decrease in cardiac TAG accumulation that was associated with both increased lipolysis and reduced lipogenesis^35^. Ceramides are a class of sphingolipids that act as lipotoxins and are therefore associated with pathophysiology phenotypes^36,37^. Inhibiting ceramide degradation by knockdown of ceramidase or sphingosine kinase in the fat body increases TAG, ceramides, and cardiac pathophysiology phenotypes^38^. Dysregulation of fat body lipid storage also leads to systemic effects on Drosophila insulin signaling, growth, reproduction, immunity, glucose homeostasis, and metabolic rate^20,29,39–41^.

Previous studies in our lab found that chronic HSD feeding produced an increase in DAGE, a class of ether lipids that has not previously been associated with lipotoxicity^34^. In this work, we explored the link between DAGE and HSD-associated pathophysiology phenotypes. Our approach combines genetics, biochemistry, and physiology to characterize the role of ether lipids in T2D-like pathophysiology phenotypes in a Drosophila model of overnutrition. We targeted the formation of DAGE through a knockdown of the gene encoding the peroxisomal enzyme dihydroxyacetonephosphate acyltransferase (*DHAPAT*) that catalyzes the formation of 1 acyl-3-glycerone phosphate, a precursor to many ether lipids, in the fat body. We found that fat body reduction of *DHAPAT* reduces DAGE and improves pathophysiology phenotypes, including obesity, glucose levels, cardiac function, and insulin signaling. Reducing *DHAPAT* seems to function by eliciting a switch from lipogenesis to beta-oxidation in animals undergoing overnutrition.

## Results

### DHAPAT promotes DAGE biosynthesis in HS-fed flies

**Figure 1:**
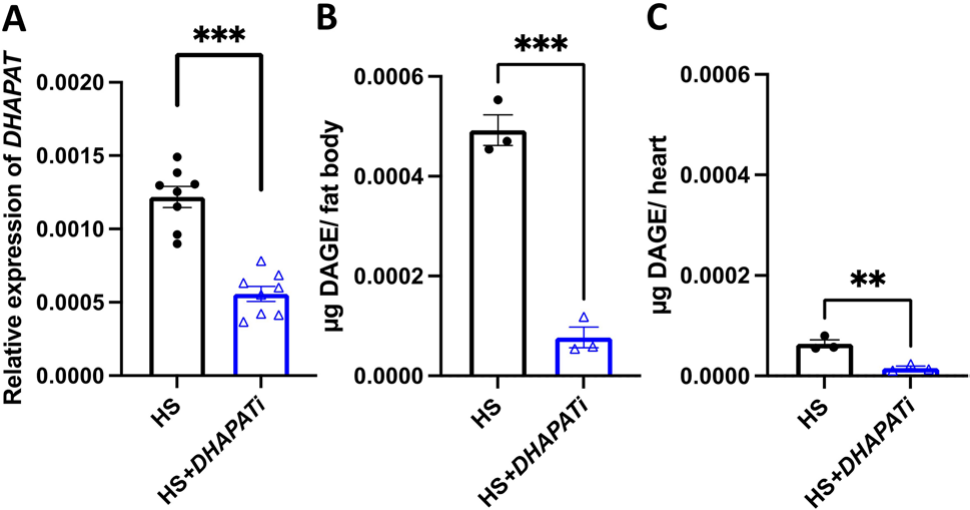
Knockdown of *DHAPAT* in the fat body reduces the putative lipotoxins DAGE. (**A**) RT and qPCR revealed a decreased expression of *DHAPAT* in the RNAi knockdown fat bodies, compared with HS-fed controls. Significance was determined by a paired two-tailed *t*-test of the 2^−ΔCt^ (n = 8). For (**B**) and (**C**), UHPLC-MS/MS was used to quantify the abundance of DAGE in (**B**) fat bodies and (**C**) hearts. Significance for (**B**) and (**C**) was determined by a two-tailed Student’s *t*-test (n = 3). The error bars represent SEM, and **p < 0.01 and ***p < 0.001 for all panels.

Because DAGE markedly accumulate in the fat body and heart after HS feeding^9^, we hypothesized that decreasing the incorporation of fatty acids into these ether lipids could reduce lipotoxicity and thereby improve pathophysiology phenotypes in flies fed a HSD. These studies focused on the fat body, the major site of lipid metabolism and therefore a likely source of toxic lipids^42,43^. To reduce the amount of DAGE, we targeted an enzyme in the ether lipid biosynthetic pathway, DHAPAT*,* via RNA interference in the fat body by combining the *r4-GAL4* driver and a *UAS-CG4625*^*RNAi*^ transgene, hereafter called “*DHAPATi*.” The control genotype was a cross between the driver and genetic background genotype. Control and *DHAPATi* larvae were reared on a 0.7 M diet and aged for 3 weeks as adults on a 1 M (34% sucrose) diet to produce chronic caloric overload, called “HS” and “HS + *DHAPATi.*” The efficacy of the knockdown was confirmed via qPCR, where there was a 43% decrease in the expression of *DHAPAT* in genetically manipulated fat bodies when compared to the control genotype (Fig. 1A). To ask whether the decrease in expression was correlated with decreased pathway output**,** we quantified the abundance of DAGE^9^. There was at least a 75% decrease in DAGE in both the fat body (Fig. 1B) and the heart (Fig. 1C), consistent with the expected role for DHAPAT in DAGE biosynthesis. This genotype was therefore used as a platform in which to explore the potential benefits of reducing DAGE in HS-fed flies.

### Fat body knockdown of *DHAPAT* influences the broader lipidome

**Figure 2:**
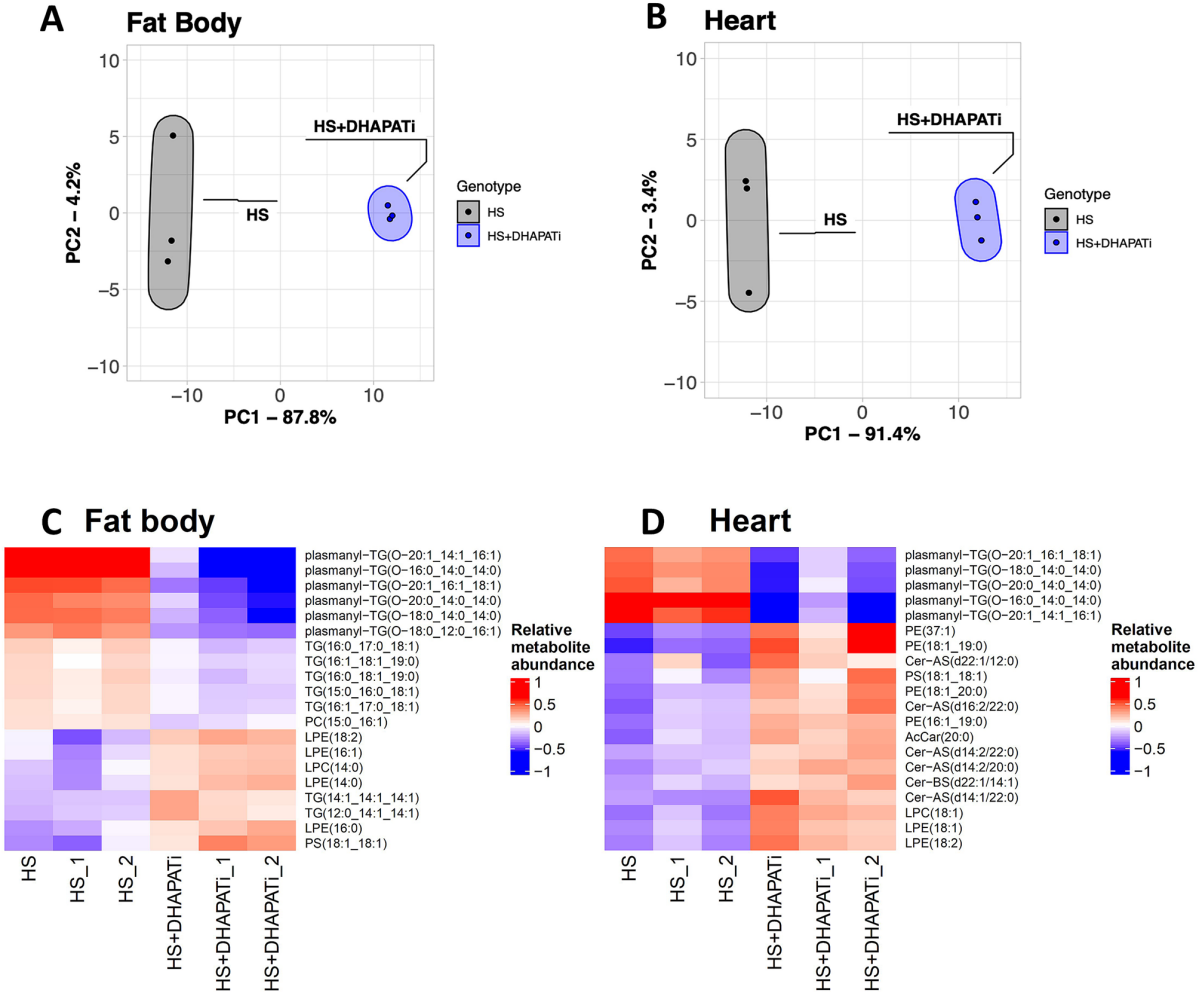
Reducing *DHAPAT* in the fat body produces a shift in the lipidome. Principle component analysis (PCA) scores plots show the variance between the lipid abundance of HS-fed control flies and HS-fed *DHAPATi* in (**A**) fat body and (**B**) heart. UHPLC-MS/MS was used to characterize the lipidome in three biological replicates (small circles) and ellipses represent a 95% confidence interval. Heat maps were generated comparing the relative fold change of metabolites between HS control and HS + *DHAPATi* for the fat body (**C**) and the heart (**D**)*.* n = 3. A Ward’s test was used to identify significantly differentially abundant lipids. Rather than set a significance threshold, shown are the top 20 from the cluster representing the highest proportion of variance in the data.

To test if reducing DHAPAT might affect other lipid pools, we used untargeted lipidomics to evaluate organs from adult flies after chronic HS feeding. Traditional UHPLC-MS/MS was used to quantify the abundance of phospholipids, glycerolipids, sphingolipids, ether lipids, and other lipid species in the heart and fat body from HS-fed control and *DHAPATi* flies. Because these organs are small and we chose a timepoint where pathophysiology (including death) is observed, many animals were combined to produce each sample and therefore, only three samples of each type were analyzed, a limitation of our study. Organs were dissected from 3-week-old flies and placed immediately into PBS on ice, then frozen at − 80 °C until extraction, separation, scanning, and quantitation. We used the statistical package Metaboanalyst V4.0 to compare lipid profiles between genotypes^44^. First, PCA scores plots were used to evaluate the variance of the lipid abundance between genotypes for both fat bodies (Fig. 2A) and hearts (Fig. 2B), using normalized peak areas as the variables. Each point represents a sample (biological replicate), and the ellipse is the 95% confidence interval. In both the heart and the fat body, there is a distinct separation of the ellipses, suggesting the metabolites vary between HS + *DHAPATi* and HS controls (Fig. 2A,B). To explore lipid classes at higher resolution, a heat map of the top 20 differentially abundant lipids was generated for both the fat body (Fig. 2C) and heart (Fig. 2D). Relative fold change between metabolites was calculated and Ward’s clustering analysis was used to determine the top 20 differentially abundant lipids. Six species of DAG ether lipids (also called plasmanyl-TG) had the highest relative reduction in fat bodies upon *DHAPAT* knockdown (Fig. 2C). There was also a decrease in the abundance of several TAG species, mostly those with 16–18 carbon acyl chains, but an increase in shorter chain (C12–C14) TAG along with an increase in numerous lysophospholipids, in *DHAPATi* fat bodies (Fig. 2C). Interestingly, the *DHAPATi* heart mimicked the fat body’s reduction in DAGE, recapitulating those five most reduced lipids (Fig. 2D). Other differentially abundant lipids were increased in the heart, including phospholipids, lysophospholipids, and both alpha-hydroxylated and canonical, long-chain ceramides (Fig. 2D). These data suggested that *DHAPATi* affected not only the fat body, but also whole animal lipid homeostasis.

### Fat body triglyceride storage is reduced by *DHAPAT* knockdown

**Figure 3:**
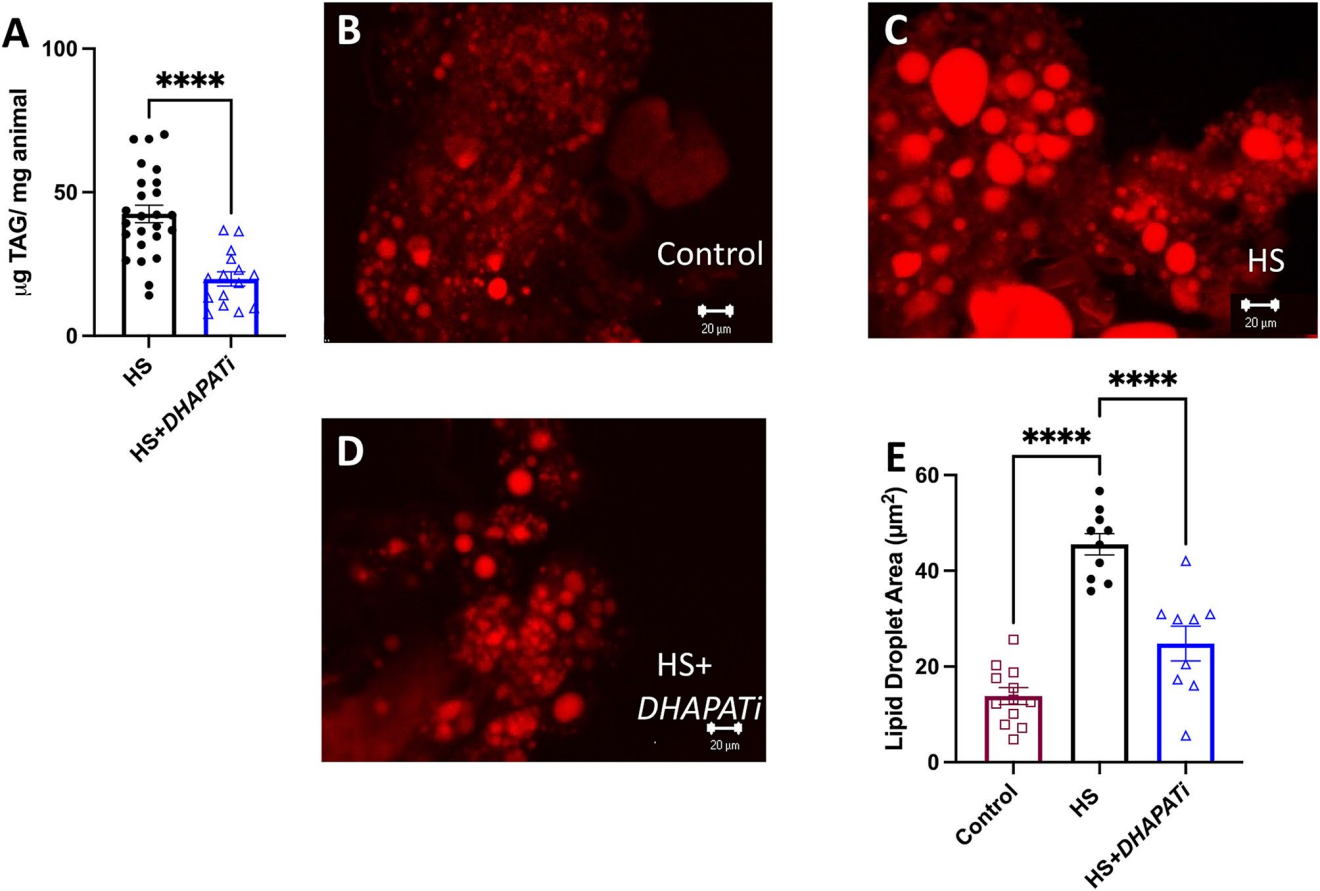
Knockdown of *DHAPAT* reduces lipid storage. (**A**) TAG decreased significantly compared to the HS-fed control (HS). Significance was determined by a two-tailed Student’s *t*-test (n = 15). (**B**–**E**) Compared to the HS-fed control genotype (HS), HS-fed *DHAPAT* knockdown decreases the size of fat body lipid droplets. Significance was determined using a one-way ANOVA and Dunnett’s multiple comparisons (n = 12). Error bars represent the SEM. *p < 0.05, **p < 0.01, ***p < 0.001. Scale bars are 20 microns.

Because DHAPAT controls the incorporation of fatty acids into complex lipids and we saw changes in the lipidome, we examined lipid storage in HS + *DHAPATi* flies. First, we measured whole animal TAG using an enzymatic TAG assay. Knockdown of *DHAPAT* in the fat body reduced overall TAG content, compared with the control genotype (Fig. 3A). This is consistent with the lipidomics data in Fig. 2C and previous work showing that TAG with longer acyl chains are the predominant forms in the fat body^20^. To test whether lipid storage was affected in the fat body in an autonomous manner, we quantified lipid droplet size after Nile Red staining. Three weeks of HS feeding produced a significant increase in fat body lipid storage droplet size in the control genotype (Fig. 3B,C,E). We observed that HS + *DHAPATi* fat bodies had smaller lipid droplets, compared to diet-matched control fat bodies (Fig. 3C,D,E). Therefore, DHAPAT has a tissue-autonomous role in lipid storage in the fat body.

### High-sugar-induced pathophysiology phenotypes were improved by knockdown of *DHAPAT*

**Figure 4:**
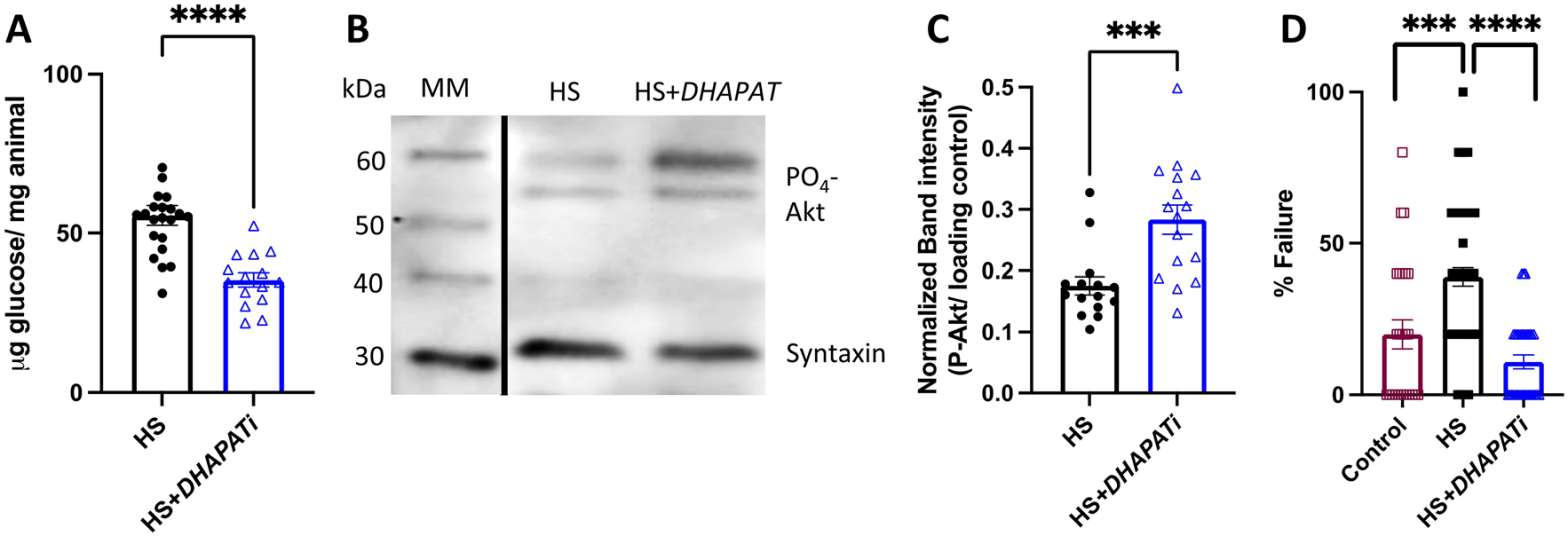
Pathophysiology phenotypes in HS-fed flies are ameliorated by *DHAPAT* knockdown. (**A**) Glucose decreased in HS + *DHAPATi*, compared to HS-fed control flies (n = 15–20). (**B**) Representative Western blot of phosphorylated Akt revealed a (**C**) increase in P-Akt in HS + *DHAPATi* fat bodies, compared to HS-fed controls (n = 16). Significance was determined by a two-tailed Student’s *t-*test. Uncropped version of western blot can be found in Supplemental Fig. 3. (**D**) Cardiac pacing-induced failure was evaluated in the HS-fed control genotype (HS) flies and compared with the same flies on a control diet (Control), and HS-fed *DHAPATi*. Significance for (**D**) was determined by a 2X2 chi-squared contingency test (n = 210–345). For all panels, error bars represent the SEM. ***p < 0.001, ****p < 0.0001.

Because HS + *DHAPATi* had decreases in stored lipids, we sought to evaluate if other obesity-associated pathophysiology phenotypes were also improved. To test whether these apparent improvements in lipid storage were associated with an improvement in carbohydrate homeostasis, whole animal glucose was quantified. We observed a significant decrease in glucose content in HS + *DHAPATi* flies (Fig. 4A). Because there was reduced TAG and glucose content, we assessed insulin signaling, which promotes glucose uptake and lipogenesis. Western blot analyses of fat bodies after HS feeding found increased Akt phosphorylation, which is a marker for insulin signaling, in HS + *DHAPATi* flies, compared with controls (Fig. 4B,C). To quantify heart function, cardiac failure was measured after pacing-induced stress in whole mounted flies. As expected, HS increased pacing-induced failure, compared to the control diet (Fig. 4D). Compared to the HS-fed control genotype, HS + *DHAPATi* improved cardiac resilience, with a significant decrease in pacing-induced cardiac failure (Fig. 4D). Similar phenotypes were observed when expressing a different RNAi transgene (see Supplementary Fig. S1 online) and reducing two ether lipid biosynthetic enzymes, fatty acid reductase (encoded by *wat*, also called *FAR1*) and alkylglycerone phosphate synthase (*AGPS*) also improved some HS pathophysiology phenotypes (see Supplementary Fig. S2 online). Taken together, these findings support a model where reducing ether lipid biosynthesis improves cardiac resilience, insulin signaling, and metabolic homeostasis in animals fed a HSD.

### The metabolic rate is increased by reducing *DHAPAT*

**Figure 5:**
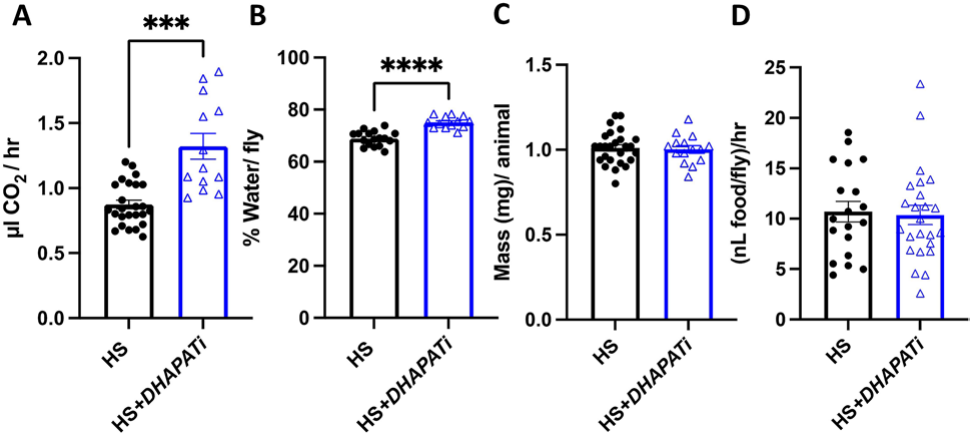
Fat body-specific knockdown of *DHAPAT* increases the metabolic rate. (**A**) Per-fly CO_2_ production and (**B**) water content were increased in HS + *DHAPATi* flies, compared with HS-fed controls (HS) (n = 13–17). (**C**) Weights were not significantly different (n = 15–20). (**D**) A consumption assay revealed no significant difference in the amount of consumed food between the HS-fed control (HS) and *DHAPATi* knockdown flies (n = 20–22). All results show the mean and error bars represent SEM. Significance was determined by a two-tailed Student’s *t*-test ***p < 0.001 and ****p < 0.0001.

Next, we explored the mechanisms by which *DHAPATi* might improve physiology on a HSD. Ether lipids make up less than 0.5% of the lipidome^9^ and a number of other metabolites were affected in *DHAPATi* flies, so it is likely that additional pathways are affected, rather than merely a stoichiometric amelioration of lipotoxin accumulation. Because of the reduction in fat body lipid storage despite increased fat body insulin signaling, we hypothesized that knockdown of *DHAPAT* would increase catabolic activity. Therefore, we measured metabolic rate by quantifying CO_2_ production using closed-flow respirometry^45^. This approach showed that HS + *DHAPATi* flies had an increased metabolic rate when compared to HS controls (Fig. 5A). If the increase in metabolic rate were due to increased mitochondrial respiratory chain activity, we would expect to see increased water content^46^. Therefore, we estimated the water content of HS + *DHAPATi* by comparing fly mass before and after desiccation and found that HS + *DHAPATi* flies contained 7% more water than HS-fed controls (Fig. 5B) with no difference in mass (Fig. 5C). In agreement with the idea that HS + *DHAPATi* flies improve metabolic homeostasis via increased catabolic activity, we found that feeding did not depend on the genotype (Fig. 5D), suggesting that decreased feeding is not the reason why HS-fed *DHAPATi* flies are lean. These findings suggest that reducing *DHAPAT* may improve animal physiology during overnutrition by increasing energy catabolism.

### Bezafibrate recapitulates the amelioration of HS-induced pathophysiology phenotypes

**Figure 6:**
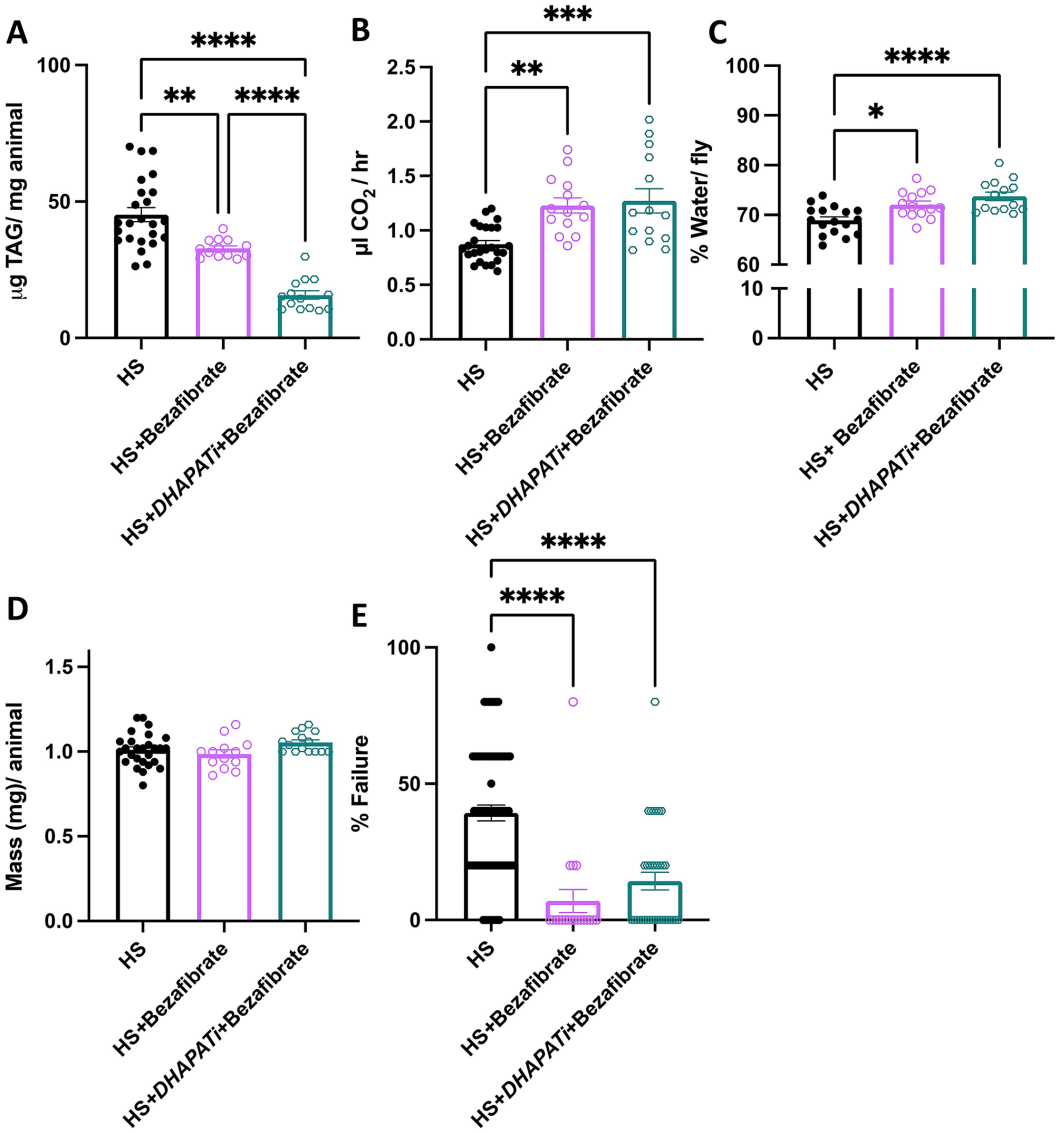
Supplementation with the PPARα activating drug, bezafibrate, improves pathophysiology phenotypes in HS-fed flies. (**A**) Effects of bezafibrate on TAG in HS-fed control and DHAPATi flies (n = 15–17). (**B**, **C**) Bezafibrate increased biomarkers of beta-oxidation, although CO_2_ production (**B**) and water content (**C**) did not significantly differ between HS + *DHAPATi* + bezafibrate and HS + bezafibrate alone (n = 15). (**D**) Mass remained unchanged between HS + *DHAPATi* + bezafibrate and bezafibrate alone (n = 15–17). Significance for (**A**–**D**) were determined by an ANOVA and Tukey test for multiple comparisons where flies were compared with HS-fed controls (HS). (**E**) Cardiac pacing-induced failure was improved by bezafibrate supplementation of the HS diet, with no significant difference between HS + *DHAPATi* + bezafibrate and bezafibrate alone. Significance for (**E**) was determined by a 2X2 chi-squared contingency test (n = 100–175). Error bars represent the SEM **p < 0.01, ***p < 0.001, ****p < 0.0001.

To explore pathways that might increase the catabolic rate in *DHAPATi* flies, we compared their phenotypes to those of flies fed the PPARα agonist bezafibrate. Previous literature demonstrated that impairing the ether lipid biosynthesis enzyme PexRAP induced beta-oxidation via PPARα in mice; therefore, we hypothesized that *DHAPATi* might act via a similar mechanism^47^. The HSD was supplemented with 0.4 µM bezafibrate, a concentration chosen based on efficacy in previous Drosophila studies^48^, to test its effects on the metabolic rate and obesity-associated pathophysiology phenotypes. As for HS + *DHAPATi*, bezafibrate significantly reduced whole animal TAG, compared to the HS-fed flies alone, similar to its effects in mammals (Fig. 6A^47^). Feeding bezafibrate to HS + *DHAPATi* flies gave TAG levels (Fig. 6A) that were not significantly different compared to *DHAPATi* alone (see Fig. 3A; 19.8 vs. 15.6 μg TAG/mg animal *p* = 0.65), suggesting they act in the same pathway. In accordance, metabolic rate was no different between *DHAPATi* fed bezafibrate and bezafibrate alone (Fig. 6B). Similar results were observed for water content (Fig. 6C), mass (Fig. 6D), and heart failure (Fig. 6E). These findings, where bezafibrate and *DHAPATi* have a non-additive effect on pathophysiology phenotypes, support a model where the knockdown of *DHAPAT* leads to an increase in beta-oxidation via a PPARα-like protein.

## Discussion

In this study, we used HS-fed Drosophila to explore the role of the ether lipid biosynthesis gene, *DHAPAT*, in obesity-associated pathophysiology phenotypes. Fat body knockdown of *DHAPAT* not only reduced DAGE, but also reduced TAG and glucose and therefore improved cardiac resilience, insulin signaling, and metabolic homeostasis in HS-fed flies, benefits that seemed to result from increased catabolic activity. The benefits to HS-fed flies with reduced *DHAPAT* were phenocopied by bezafibrate, a drug that promotes lipolysis and fatty acid beta-oxidation in mammals.

Reducing lipid storage is associated with improved pathophysiology in some^49,50^ but not all^5,51^ overnutrition models. In some contexts, lipid storage can be a safe sink that prevents fatty acids from meeting more toxic fates. Lipodystrophic patients who cannot store fat, for example, develop extreme insulin resistance and other pathophysiology phenotypes^52^. In the current study, *DHAPATi* produced a decrease in lipid storage along with improvements in pathophysiology phenotypes, consistent with a context where leanness is beneficial (Fig. 3). Reducing lipid storage might help by pushing free fatty acids toward catabolism or into beneficial lipid fates instead, or could confer an advantage to the animal simply by freeing up cellular space or by providing resources like ATP. These pathways are candidates of interest for future study.

One mechanism by which cells can reduce lipid storage is to increase fatty acid degradation via mitochondrial beta oxidation. We hypothesize that systemic improvement of pathophysiology phenotypes after knockdown of *DHAPAT* could be due to improved oxidative activity. We saw that HS-fed *DHAPATi* had an increase in respiration and water content, suggesting increased electron transport along the mitochondrial respiratory chain (Fig. 5). Improvements in T2D-like physiology have been observed in many model systems when mitochondrial fat catabolism is increased. Of course, exercise activates this pathway and improves a range of overnutrition-associated comorbidities^53^. Fibrate drugs have been used to pharmacologically stimulate fatty acid oxidation and they provide similar therapeutic benefits^54^. Other approaches that increase the oxidative capacity of mitochondria can also improve T2D-like pathophysiology phenotypes. Feeding chemical uncouplers of the mitochondrial proton gradient increased lipid catabolism and improved obesity-associated pathophysiology^55,56^. Genetic alterations that increase mitochondrial oxidative capacity also improve physiology in a number of metabolic disease models. Increased expression of the mitochondrial uncoupling proteins potentiates fatty acid oxidation and improves pathophysiology in T2D models^57–59^. Knockout mice lacking acetyl-CoA carboxylase 2 (ACC2) exhibit reduced cellular malonyl CoA, an inhibitor of the mitochondrial carnitine shuttle system, which imports fatty acids for catabolism. Mouse *ACC2* mutants display increased fatty acid oxidation and insulin sensitivity when fed a high-fat or high-sugar diet^60,61^. Thus, the regulation of fatty acid flux is important for protecting against T2D-like pathophysiology. Supplementing the HSD with bezafibrate had similar benefits to, but did not improve phenotypes in, *DHAPATi* flies. This suggests that a bezafibrate target, likely a nuclear receptor, exists in Drosophila. These data also fit a model where HS-fed *DHAPATi* flies already have increased lipid catabolism, compared to controls, such that bezafibrate cannot provide an additional benefit at a concentration that is therapeutic in control flies. It is possible that reducing DAGE promotes respiration as a byproduct of changes in gene expression, increased demand for activated energy carriers, uncoupling of the mitochondrial proton gradient, and/or the state of the lipidome in *DHAPATi* flies.

Increased oxidative capacity might be expected to arise from changes in ether lipids. Reducing ether lipids in mice led to the activation of the transcription factor PPARα, which promotes the expression of genes encoding enzymes that function in lipid catabolism^16,62^. A murine knockdown and mutant of *PEXRAP* (peroxisomal reductase activating PPARγ), an ether lipid biosynthetic enzyme downstream of DHAPAT, showed increased expression of PPARα targets^47^. Like *DHAPATi* flies (Figs. 3, 4), *PEXRAP* knockdown and mutant mice have decreased TAG content and increased insulin sensitivity^47,62^ To test if activation of a PPARα-like pathway was a possible mechanism for *DHAPATi* improvement of HS-induced pathophysiology, we supplemented the HSD with bezafibrate, a PPARα agonist. These findings are consistent with a model where increased fatty acid oxidation benefits HS-fed flies and they further suggest that a lipid that is increased in *DHAPATi* flies may be acting as a ligand for a PPARα-like factor.

Taken together, our findings support a model where overnutrition produces T2D-like pathophysiology in an ether lipid-dependent manner in flies. Reducing *DHAPAT* expression produced a metabolic and functional shift that improved pathophysiology phenotypes and seemed to favor fat oxidation over fat storage. This represents a novel platform in which to understand the molecular underpinnings of the pathways and metabolites that contribute to diet-induced lipotoxicity.

## Methods

### Fly lines and husbandry

Control flies, *w*^*1118*^ (VDRC stock #60000) and the TRiP insertion site control genotype (BDSC stock #36304)*, DHAPAT* RNAi fly stock lines (VDRC stock #1429 or BDSC stock #52914), *wat/FAR1* RNAi (VDRC #1333), and *AGPS* RNAi (VDRC #3321) were obtained from the Vienna Drosophila Resource Center^63^ or the Bloomington Drosophila Stock Center^64^. Fly stocks were reared with controlled humidity on a 12/12 h day-night cycle with a 5% dextrose-cornmeal-yeast-agar medium diet. *UAS*-RNAi lines were crossed with *UAS-Dcr2; r4-GAL4* to drive expression and enhance knockdown in the fat body. Experimental crosses were reared on 0.7 M (24% sucrose) or 0.15 M (5% sucrose) food and within 24 h of eclosion flipped onto 1 M (34% sucrose) or 0.15 M food and aged for 3 weeks. Diets are as described^21^. Flies were flipped onto new food every 3 days for the periods of aging.

### Tissue collection

For all dissections, flies were anesthetized with FlyNap (trimethylamine) and organs isolated by fine dissection^65^. Fat bodies and hearts were collected after a ventral filet of the organism, when the fat body was removed from the cuticle by fine dissecting scissors and the heart was removed using jeweler’s forceps. For quantitative assessments, the number of organs was defined as the total number of organisms that tissue was collected from, and then normalized to the amount of sample loaded.

### Whole animal TAG and glucose assays

Five, 3-week-aged, female flies were homogenized in PBS + 0.1% Tween. Samples were heated at 65 °C for 5 min^21^ and 2 µl of the homogenate were added to either 198 µl of Infinity triglyceride reagent (Thermofisher Scientific, Waltham, MA, USA, # TR22421) or 198 µl of the Infinity glucose reagent (ThermoFisher # TR15421) and analyzed with a VersaMax microplate spectrometer (Molecular Devices, San Jose, CA, USA) at 540 nm (TAG) or 340 nm (glucose) after a 5-min incubation at 37 °C. An ANOVA or a two-tailed student’s *t*-test was used to determine significance, depending on the number of comparator groups, using GraphPad’s Prism software (V. 9.2).

### Western blots

Fifteen, 3-week-old, female fat bodies were dissected and frozen in sample buffer at − 80 °C before immunoblotting. Samples were run on Stain-Free gels (Bio-Rad, Hercules, CA, USA #456–8126) for total protein quantification. Western blotting compared p-AKT in HS-fed *DHAPATi* and HS-fed control flies, with syntaxin as the loading control. Cell Signaling’s rabbit anti-Drosophila antibody (#4054) against p-AKT (Ser505) was used at a dilution of 1:500, and a U. of Iowa Developmental Studies Hybridoma Bank antibody was used against syntaxin (#8C-3, made in the mouse, 1:10,000). Secondary antibodies were from Santa Cruz (goat anti-mouse HRP #SC-2005 and goat anti-rabbit HRP #SC-2004) and used at 1:10,000. Gels and blots were imaged with a Bio-Rad Chemidoc Touch imager, then quantified with Bio-Rad ImageLab v6.0.

### Consumption assays

The consumption assay was adapted from Shell et al. Flies aged 3 weeks on a HSD were transferred to media supplemented with 2% FD&C Blue #1 for 2 h^66^. Groups of 4 flies were homogenized with a mortar and pestle and supernatants were read at a wavelength of 630 nm by the VersaMax spectrophotometer. A two-tailed Student’s *t-*test was performed to determine statistical significance using Prism software (V. 9.2).

### Cardiac pacing

3-week-old flies aged on a 1 M or 0.15 M diet were anesthetized with FlyNap and separated by sex. In groups of 5, the flies were placed on a glass slide containing aluminum stripes on either side. Conductive wires were attached to aluminum strips and then electrical shocks were administered using the square wave stimulator (Phipps & Bird, VA, USA): 50 V, 30 ms duration, 8 Hz for 30 s for RNAi lines with *w*^*1118*^*.* Heart failure was defined by the percentage of flies that had a complete cessation of a heartbeat within 30 s of the electrical shock. Chi-squared analysis was used to determine significance using Prism.

### Reverse transcription and qPCR

Twenty, 3-week-old, female fat bodies were harvested as described above in 500 µl of Ribozol (VWR; N580-100ML) and RNA was prepared per manufacturer’s instructions. RNA was treated with DNase (VWR; PIER89836) and then ran over a Qiagen RNeasy purification column (Qiagen, Germantown, MD, USA Cat #74104). Total RNA was quantified using Qubit 2.0 fluorometer HS Assay kit (Life Technologies/ThermoFisher Scientific, Waltham, MA, USA Cat. # 10210). Reverse transcription was done with BioRad iScript reagent (BioRad #1708890) using either the *DHAPAT* primers (F: CGACATCGAGATACCAGGAATAGC and R: TCATCGTTGGAGAAGCTGCG) or the ribosomal protein-coding mRNA control *rp49* primers (F: GCACTCTGTTGTCGATACCC and R: CAGCATACAGGCCCAAGAT). Each primer set extended an intron region to ensure no non-coding DNA contamination. Axygen Maxygene II benchtop thermocycler (Axygen/Corning, Corning, NY, USA) was used for reverse transcriptions. Sso advanced Sybr-green qPCR reagent (BioRad #172-5272) was added, and quantitative PCR was conducted using BioRad CFX Connect Real-Time System. Samples were normalized to *rp49* gene expression using 2^−ΔCT^ and a paired two-sample *t*-test was used to determine significance using Prism.

### UHPLC-MS/MS

One hundred, 3-week aged, female fly hearts, and 40 fat bodies were collected by fine dissection. Organs were homogenized by mechanical shearing with a 20-gauge needle in 200 µl of PBS (pH 7.4), with 190 µl extracted for UHPLC-MS/MS and 10 µl used for protein determination by a Bradford assay^65^. A Folch extraction was followed by separation on a C18 column. Untargeted LC–MS lipidomics analysis was carried out in positive and negative ion modes using a Thermo Q-Exactive Orbitrap mass spectrometer with Dionex UHPLC as described by Ulmer et al.^67^. Separation was achieved on an Acquity BEH C18 column and peaks were assigned using exact mass matching via LipidMatch^68^. Peak area was normalized to the total ion current and internal standards (Avanti Polar Lipids # 330,707) were added before extraction to determine the absolute lipid abundance. Metaboanalyst was used to calculate relative fold changes, using a Ward’s clustering analysis to generate clusters of lipid species based on the smallest combined error for sum of squares. Principal component analysis was done using Bioconductor in R version 1.4.1106, and plotted with the package ggplot^69,70^. Detailed methods, R code, and metabolomics data have been deposited at Zenodo (https://zenodo.org/) in records 6667745, 6589774, and 6585456, respectively).

### Drug administration

Bezafibrate (Thermofisher, Waltham, MA, USA, Cat.#AAJ61412-06) was dissolved in ethanol and added to the HS food at a concentration of 0.4 µM^48^. One-week-old flies fed HS were placed on the bezafibrate-supplemented food for 2 weeks before analysis.

### Lipid droplet visualization

Five 3-week aged, female flies were ventrally filleted in EGTA, guts removed, then the remaining fat body and abdominal cuticle were fixed in 4% PFA and stained with the lipophilic dye Nile Red (Thermo #AC41571) at a concentration of 0.001% in PBS for 30 min. Fat bodies were imaged from pelts mounted with VectaShield (Vector Labs, Burlingame, CA, USA, Cat. # H1000). Confocal microscopy was used to detect fluorescence at an emission wavelength of 543 nm. Five fields of view were imaged from each animal and averaged to represent droplet size from throughout this tissue. Image J was used to measure lipid droplet size using the Analyze Particles feature, where particle size was specified as 20-infinity, and average area was calculated. An ANOVA and Tukey post-hoc multiple comparisons tests were done to determine statistical significance using Prism.

### Respirometry

Metabolic rates were measured using stop-flow respirometry using a procedure adapted from Powell et al. 2020^45,71,72^. Groups of 5 flies were separated 24 h prior to experimentation. They were transferred without anesthesia into 5 mL Norm-Ject syringes (Air Tite Products, VA, USA) fitted with three-way luer valves (Cole Parmer, Vernon Hills, IL, USA). Flies were given 1 mL of atmospheric air prior to the purge along with four control syringes containing only atmospheric air. Syringes were then flushed with CO_2_ free air, using an aquarium pump that pushes air through columns with Drierite (A. Hammond Co. Xenia, OH, USA) and Ascarite II (Thomas Scientific, Philadelphia, PA, USA), and humidified with pH 4 water bubbler, and a total of 2 mL of CO_2_ free air were added to the syringe. Samples were left in the incubator for 1 h after purging. After the hour, 1 mL of air was injected into a flow-through system with a Li-Cor 7000 infrared CO_2_ analyzer (Lincoln, NE, USA) and analyzed with Expedata. An MFC-2 mass flow control unit (Sable Systems International) with a Sierra instruments (Monetary, CA, USA) mass-flow valve maintained the flow rate at 150 mL/min. The additional water added to the sample was scrubbed out using magnesium perchlorate before entering the sample cell of the gas analyzer, the system scrubbed of CO_2_ and H_2_O with another Drierite and Ascarite containing scrubber. The respiration rate was then calculated using the bolus integration method of^71^. Results were analyzed in R with a package created by Ragland et al. 2009, and modified by Powell et al. 2020^45,72^.

## Supplementary Information

Supplementary Information.

## Data Availability

All data collected are contained within this manuscript or linked via Zenodo as noted.

## Abbreviations

T2D: Type two diabetes
HSD: High-sugar diet
HS: Control genotype fed HSD for 3 weeks
*DHAPATi*: Flies expressing dsRNA targeting DHAPAT in their fat bodies
HS + *DHAPATi*: *DHAPATi* fed HSD for 3 weeks
PPAR: Peroxisomal proliferator activated receptor
TAG: Triglycerides
DAG: Diglycerides
DAGE: Diglyceride ethers
FFA: Free fatty acids
DHAPAT: Dihydroxyacetonephosphate acyltransferase
