# Supplementary material for: Reducing ether lipids improves Drosophila overnutrition-associated pathophysiology phenotypes via a switch from lipid storage to beta-oxidation
Source: Sci Rep. 2022 Jul 29;12:13021. doi: 10.1038/s41598-022-16870-4 (PMC9338069; doi:10.1038/s41598-022-16870-4)

Supplementary Information for **"Reducing ether lipids improves Drosophila overnutrition-associated pathophysiology phenotypes via a switch from lipid storage to beta-oxidation"**
Christie Santoro, Ashley O'Toole, Pilar Finsel, Arsalan Alvi, and Laura Palanker Musselman

Supplementary Fig. S1. An alternate *DHAPAT* RNAi transgene produced similar phenotypes. These studies used the Harvard TRiP insertion site genotype to generate controls and UAS-RNAi line 52914 to knock down *DHAPAT* (called *DHAPAT*i2). High-sugar controls (HS) were compared with HS-fed *DHAPATi2*. A) Whole animal TAG, compared to HS-fed control. B) Whole animal glucose, compared to HS-fed control. C) Weights of *DHAPATi2* were significantly lower than HS-fed. D) Cardiac pacing-induced failure rates after three weeks of HS feeding. Significance for A, B, and C was determined by a two-tailed Student's *t*-test (n=14-16). Significance in D was determined by a 2X2 chi-squared test (n=247-287). Error bars represent the SEM. *p<0.05, **p<0.01, *** p<0.001.

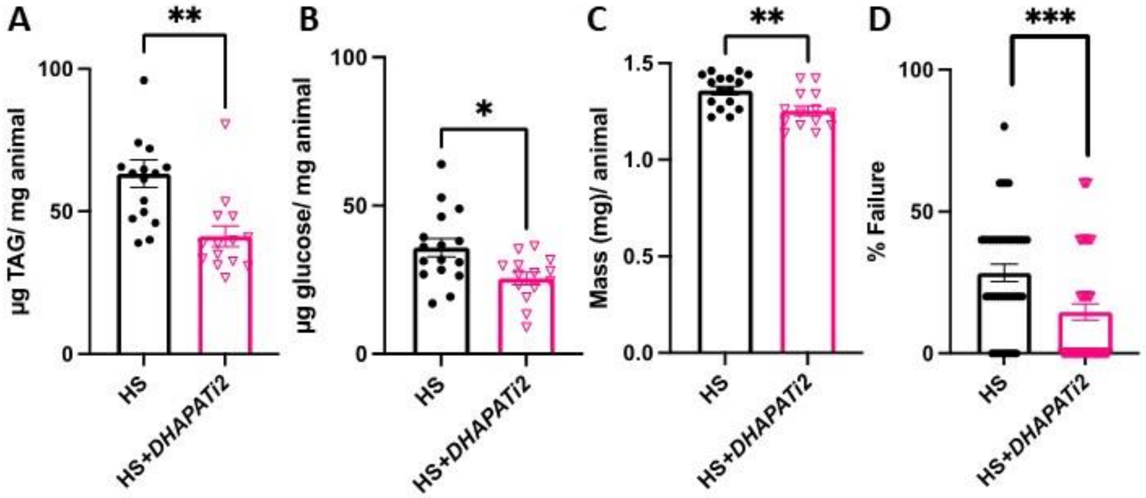

Supplementary Fig. S2. Support for a fat body role of ether lipid biosynthetic enzymes in HS pathophysiology phenotypes. Double-stranded RNA targeting two genes was expressed using the *r4-GAL4* driver. (A) Relative TAG concentrations in HS-fed control, *FAR1* and *AGPS* RNAi flies (n=12). (B) Mass decreased significantly in *AGPS* RNAi flies (n=12). Significance in A-B was determined by an ANOVA and Tukey test for multiple comparisons where flies were compared with HS-fed controls (HS). C) Cardiac pacing-induced failure was improved by *AGPS* knockdown, with no significant difference between HS controls and *FAR1* RNAi. Significance for (C) was determined by a 2X2 chi-squared contingency test (n=195-200). Error bars represent the SEM and ****p<0.0001.

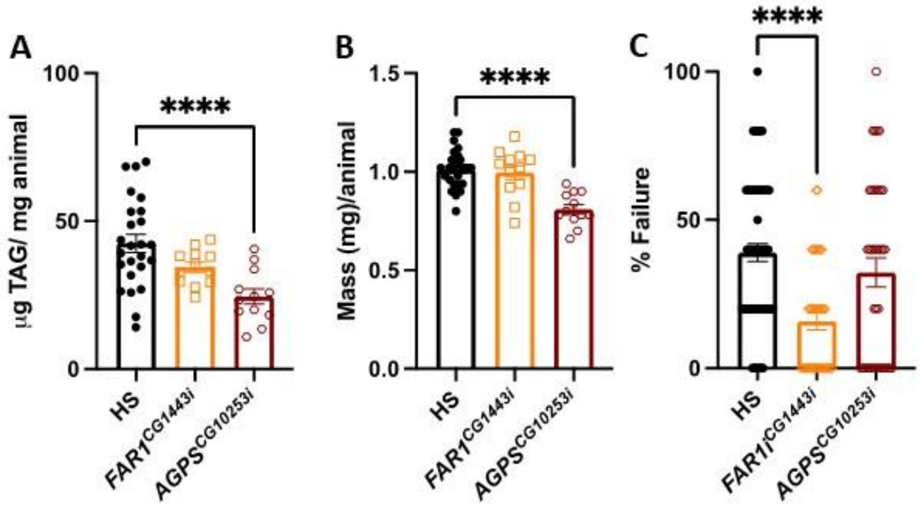

Supplementary Fig. S3. Western blot of P-Akt signaling. A) Full image of western blot of P-Akt that revealed an increase in P-Akt compared to the HS-fed control (HS) (n=16). No other editing was performed to the image.

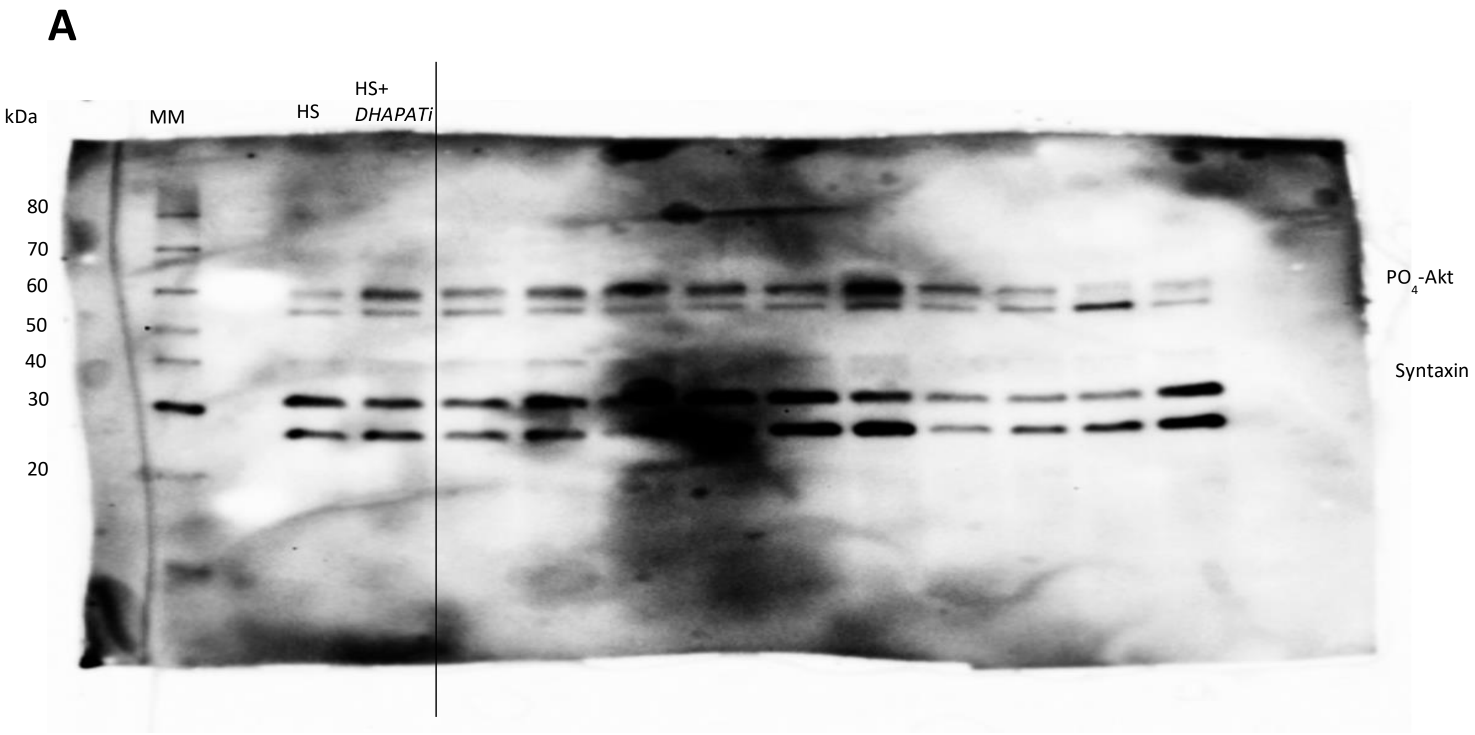

Supplement: Supplementary file 1 — Supplementary Information. [file 41598_2022_16870_MOESM1_ESM.pdf]
